# The Frequency of HLA-A, HLA-B, and HLA-DRB1 Alleles in Patients with Acute Lymphoblastic Leukemia in the Turkish Population: A Case-Control Study

**DOI:** 10.4274/tjh.2016.0102

**Published:** 2016-12-01

**Authors:** Türkan Patıroğlu, H. Haluk Akar

**Affiliations:** 1 Erciyes University Faculty of Medicine, Department of Pediatric Immunology, Kayseri, Turkey; 2 Erciyes University Faculty of Medicine, Human Leukocyte Antigen Tissue Typing Laboratory, Kayseri, Turkey

**Keywords:** Acute lymphoblastic leukemia, Human leukocyte antigen alleles, Risk groups

## Abstract

We studied the frequencies of human leukocyte antigen alleles (A, B, and DRB1) in 90 patients with acute lymphoblastic leukemia (ALL) and then compared them with 126 controls in this study. Although the frequencies of the A*03 allele, the DRB1*03 allele, the DRB1*04 allele, the A*02/B*35/DRB1*13 haplotype, and homozygosity of A*02 were higher in patients (p=0.006, p=0.003, p=0.002, p=0.01, and p=0.02, respectively), the frequencies of the A*23, B*13, B*40, and DRB1*13 alleles were lower (p=0.002, p=0.07, p=0.002, and p=0.003, respectively) in patients than controls. The frequencies of the DRB1*04 and DRB1*07 alleles were higher in patients in the high-risk group and standard-risk group, respectively (p=0.009 and p=0.007, respectively). This study indicated that the frequency of the A*03 allele, the DRB1*03 allele, the DRB1*04 allele, the A*02/B*35/DRB1*13 haplotype, and A*02 homozygosity may play a predisposing role in patients with ALL in the Turkish population. The frequency of the DRB1*04 and DRB1*07 alleles may also be associated with high risk and standard risk in patients with ALL, respectively.

## Abstract (tr)

Bu çalışmada akut lenfoblastik lösemili (ALL) hastalarda insan lökosit antijeni alellerinin (A, B, ve DRB1) Türk toplumundaki dağılımını araştırdık. Çalışmaya 90 ALL hastası ve 126 sağlıklı kontrol dahil edildi. Kontrollerle karşılaştırdığımızda ALL hastalarında A*03, DRB1*03, DRB1*04 alellerinin, A*02/B*35/DRB1*13 haplotipinin ve homozigot olarak A*02 alelinin daha sık olarak (sırası ile p=0,006, p=0,003, p=0,002, p=0,01 ve p=0,02) dağıldığını gözlemledik. Aksine A*23, B*13, B*40 ve DRB1*13 alelleri (sırası ile p=0,002, p=0,07, p=0,002 ve p=0,003) ise kontrol grubunda daha fazla olarak saptandı. Ayrıca DRB1*04 ve DRB1*07 alelleri (sırası ile p=0,009 ve p=0,007) risk gruplarına göre yapılan karşılaştırmada sırası ile yüksek ve standart riskli hastalarda daha fazla bulundu. Bu çalışma ile ALL hastalarında Türk toplumu için A*03, DRB1*03, DRB1*04 alelleri, A*02/B*35/DRB1*13 haplotipi ve homozigot formdaki A*02 alelinin bir risk faktörü olabileceği gözlemlendi. Ayrıca DRB1*04 ve DRB1*07 alellerinin risk gruplarının oluşmasında sırası ile yüksek ve standart risk gruplarında daha fazla bulunabileceği tespit edildi.

## INTRODUCTION

Acute leukemia is an uncontrolled clonal disease due to the increasing of immature hematopoietic cells with a rate of at least 25% in the bone marrow [1]. Acute lymphoblastic leukemia (ALL) is the most common cancer in pediatric populations [2]. The incidence of ALL is about 30 cases per million persons younger than 20 years. It is also the most common cause of death among cancers in children [3,4]. Patients with ALL can be classified into 3 risk groups as follows: a standard-risk group (SRG), a moderate-risk group (MRG) with adequate early treatment response, and a high-risk group (HRG) with inadequate response to induction treatment or Philadelphia chromosome-positive ALL [4,5]. Human leukocyte antigen (HLA) genes encode cell surface glycoproteins associated with antigen presentation that selectively interact with short peptide fragments derived from non-self and self-proteins. The HLA class I molecules (A, B, and C) present intracellular antigens to CD8+ T cells, while class II molecules (DR, DQ, and DP) present extracellular antigens to CD4+ T cells, which activate macrophages and B cells. HLA has a major role in regulating host responses to infections. It has been hypothesized that the HLA alleles may have an important role in predisposal to ALL [6]. The HLA genes are the most polymorphic genes in the human genome [7]. An association between ALL and HLA alleles has been shown in the literature; however, the data are not conclusive so far [8,9,10,11,12,13]. There are no identified consistent leukemia-associated HLA class I genes to date, but investigations of HLA class II genes such as DRB3, DRB4, and DRB5 have demonstrated consistent associations in patients with leukemia [14]. Genome-wide association studies have also identified some other risk alleles in different genes such as CDKN2A, PIP4K2A, GATA3, ARID5B, and CEBPE in patients with ALL [15]. In this study, we aimed to evaluate the association of HLA alleles, haplotypes, and homozygosity in patients with ALL.

## MATERIALS AND METHODS

### Study Population

This retrospective study was performed at the HLA Tissue Typing Laboratory of Erciyes University in Kayseri, Turkey. Ninety pediatric ALL patients (58 male patients and 32 female patients, 76 patients with B-cell and 14 patients with T-cell ALL, aged 7 months to 16 years) and 126 age- and sex-matched unrelated healthy controls (72 males and 54 females, aged 1-18 years) were enrolled in this study, all of Turkish ethnic origin. Participants in the control group were selected from among unrelated healthy donors who were studied for HLA alleles for transplantation (for solid organ or hematological malignancies). In the 90 patients with ALL, risk groups were as follows: 29 patients in the SRG, 37 in the MRG, and 24 in the HRG.

### Human Leukocyte Antigen Typing

Whole venous blood specimens were collected in 2KE tubes with EDTA for all participants. Genomic DNAs were obtained using the BioRobot EZ1 (QIAGEN, Hilden, Germany). Genotyping of HLA alleles was done as low-resolution typing by the polymerase chain reaction with sequence-specific oligonucleotide probe (PCR-SSOP) method (Gen-Probe Lifecodes, Stanford, CA, USA). MATCH IT DNA software version 1.2.0 was used for HLA allele interpretation.

### Statistical Analysis

Statistical analyses were performed using SPSS 22. The association of alleles, haplotypes, and homozygosity was compared with the chi-square test (χ^2^). Two groups were in accordance with Hardy-Weinberg equilibrium (p>0.005). The Bonferroni correction test was performed for multiple comparisons in risk groups. A value of p≤0.05 was accepted to be statistically significant.

## RESULTS

The frequencies of A, B, and DRB1 alleles are shown as 2n in Tables 1, 2, and 3. Although the frequencies of the A*03, DRB1*03, and DRB1*04 alleles were observed to be higher (p=0.006, p=0.003, and p=0.002, respectively) in patients with ALL, the frequencies of A*23, B*13, B*40, and DRB1*13 (p=0.002, p=0.07, p=0.002, and p=0.003, respectively) were observed to be lower. In the second step, we evaluated the frequency of haplotypes (Table 4). The A*02/B*35/DRB1*13 haplotype was found to be higher in frequency in patients with ALL (7.8% vs. 0.8%, p=0.01; Table 4). In the third step, we investigated the homozygosity of HLA alleles (Table 5). The most homozygous alleles were A*02 (6.7% vs. 0.8%, p=0.02) and DRB1*11 (6.7% vs. 4%). The frequency of HLA alleles was compared among patients according to risk groups in the last step (Table 6). Although DRB1*04 frequency was observed to be higher in patients in the HRG (p=0.009), DRB1*07 frequency was found to be higher in patients in the SRG (p=0.007).

## DISCUSSION

The underlying mechanisms are not well defined in patients with ALL [1,16]. The presence of genetic effects on the development of leukemia was observed in monozygotic twins [16,17]. Some studies have shown that some HLA alleles may be involved in the development of leukemia [14,17]. The first HLA association was reported in 1967, with increased frequency of the A*02 allele in patients with ALL [18]. On this topic, however, the data remain insufficient. Several associations have been reported between leukemia and HLA genes such as DRB3, DRB4, and DRB5 so far [14]. There are some inconsistencies among studies in the literature. The frequency of DRB1*13 as a protective allele was reported to be lower in some previously reported studies, as it was in our study [10,12]. This similarity for the DRB1*13 allele among studies may be explained by geographic proximity and interactions between Iranian [10] and Turkish populations [12]. In another Turkish study, the frequency of DRB1*04 was reported to be higher and the frequency of A*23 was reported to be lower in patients with ALL, as in our study [11]. In that study, inconsistent with our data, B*07 frequency was observed to be lower in patients with ALL. In another Turkish study, a positive association was reported in some alleles such as A*11 and DRB1*01, which is inconsistent with our results in patients with ALL [12]. These discrepancies among Turkish studies may result from the size of study populations. In this study, we also observed a positive association with A*03, DRB1*03, and DRB1*04 alleles in patients with ALL. In contrast to our study, Fernandes et al. [19] reported a negative association between ALL and DRB1*04 in an adult population. Our results contribute some new information to the literature about HLA associations in patients with ALL for the Turkish population. For example, the frequency of A*03, B*13, B*40, and DRB1*03 was inconsistent with the results of other reported Turkish studies [11,12]. In the literature, some HLA haplotypes have also been accepted as important risk factors for developing leukemia [10,19]. For example, a negative association with the A*02/B*35/DRB1*13 haplotype was observed in patients with ALL [12]. On the contrary, A*02/B*35/DRB1*13 haplotype frequency was observed to be higher in our study as a predisposing factor. Homozygosity of DRB4*01 was also reported to be a risk factor in children with leukemia [20]. In this study, the homozygosity of A*02 was observed to be higher in patients as a predisposing factor. In the last step of our research, although the frequency of DRB1*04 was observed to be higher in patients with high risk, the frequency of the DRB1*07 allele was found to be higher in patients with standard risk. As a limitation, the number of participants in our study was not large enough to make conclusive decisions about HLA association, which may lead to some discrepancies from other Turkish studies of patients with ALL. Additionally, some odd ratios (OR) in this study were calculated as lower than zero (<0.00), such as those for DRB1*03 (OR=0.36), DRB1*04 (OR=0.50), the A*02/B*35/DRB1*13 haplotype (OR=0.09), and A*02/A*02 homozygosity (OR=0.11). The lower OR values can most likely be explained by the small importance of these data among the genetic factors predisposing to ALL.

In conclusion, although A*03, DRB1*03, and DRB1*04 were observed to be susceptible alleles, A*23, B*13, B*40, and DRB1*13 were found to be protective alleles in patients with ALL. Although some results of our study support earlier findings, others are inconsistent. The increasing frequency of DRB1*04 and the decreasing frequency of A*23 and DRB1*13 alleles support results of earlier Turkish studies [11,12]. As new data, the frequencies of the A*02/B*35/DRB1*13 haplotype and A*02 homozygosity were observed to be higher as predisposing factors in patients with ALL. The frequency of DRB1*07 and DRB1*04 was observed to higher in the SRG and HRG, respectively, as additional predisposing factors.

## Ethics

Ethics Committee Approval: Retrospective study; Informed Consent: It was not required.

## Figures and Tables

**Table 1:**
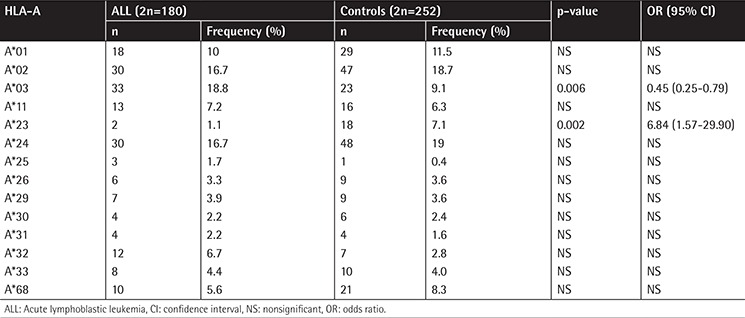
The frequency of HLA-A alleles.

**Table 2:**
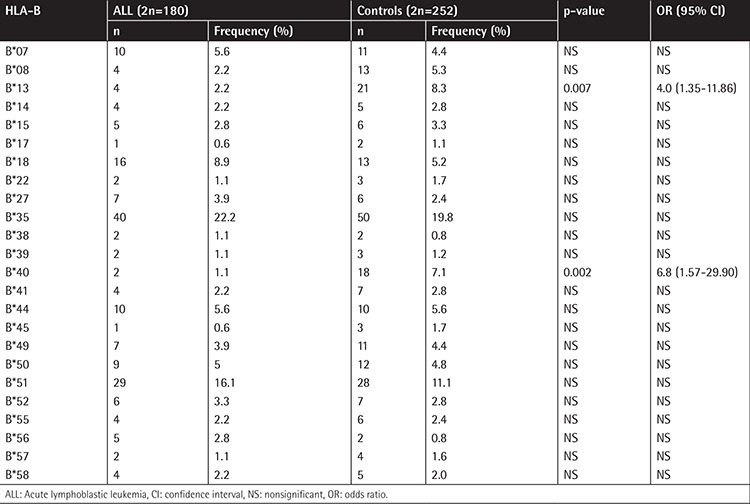
The frequency of HLA-B alleles.

**Table 3:**
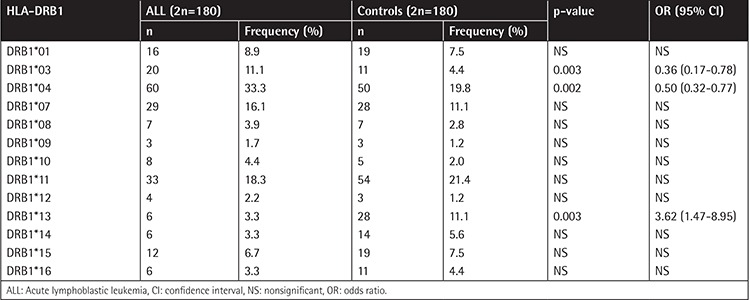
The frequency of HLA-DRB1 alleles.

**Table 4:**
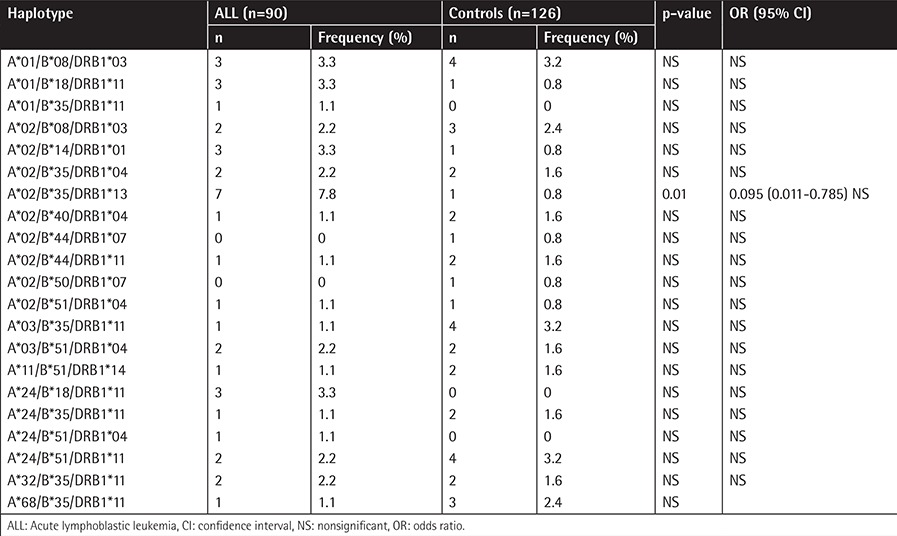
The frequency of HLA-A, -B, and -DRB1 haplotypes.

**Table 5:**
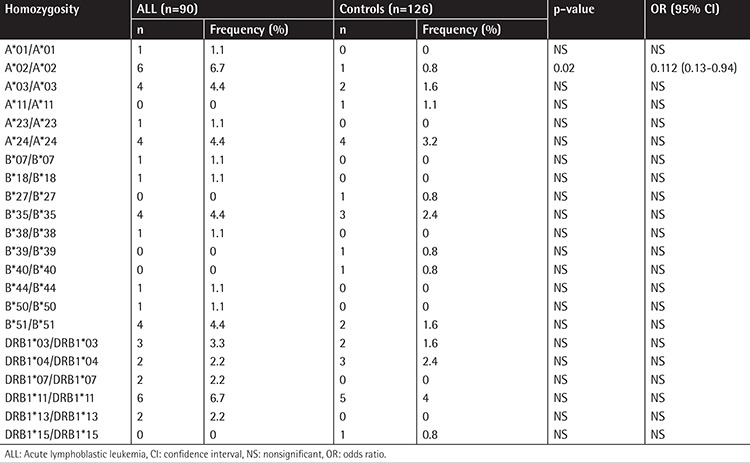
The homozygosity of HLA alleles.

**Table 6:**
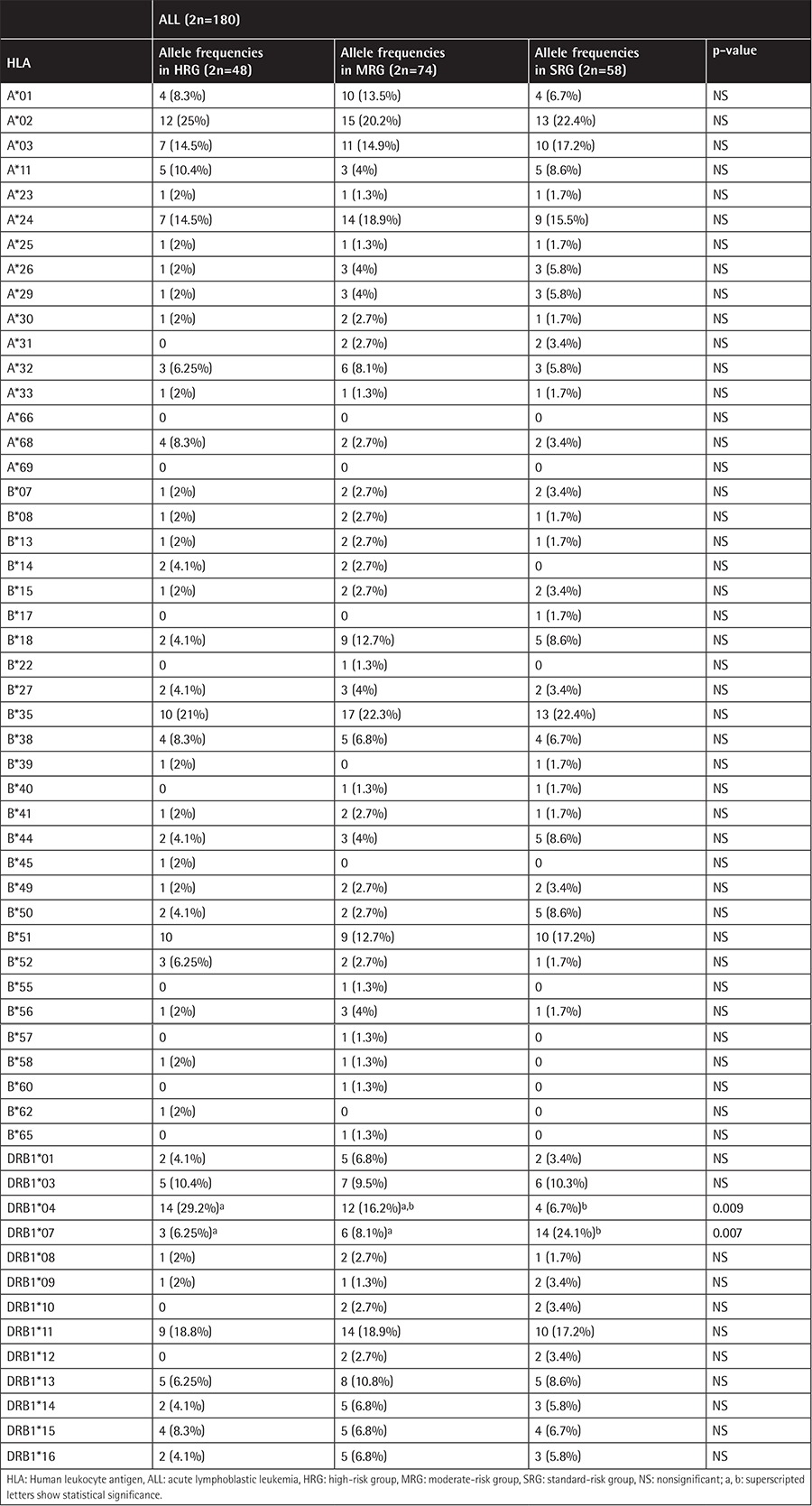
The frequency of HLA alleles in risk groups.
